# Speckle tracking stress echocardiography in children: interobserver and intraobserver reproducibility and the impact of echocardiographic image quality

**DOI:** 10.1038/s41598-018-27412-2

**Published:** 2018-06-15

**Authors:** Lucia Wilke, Francisca E. Abellan Schneyder, Markus Roskopf, Andreas C. Jenke, Andreas Heusch, Kai O. Hensel

**Affiliations:** 1 HELIOS University Medical Center Wuppertal, Children’s Hospital, Center for Clinical & Translational Research (CCTR), Witten/Herdecke University, Witten, Germany; 2 EKO Children’s Hospital, Witten/Herdecke University, Oberhausen, Germany; 3 University of Cambridge, Addenbrooke’s Hospital, Department of Paediatrics, Cambridge, UK

## Abstract

Speckle tracking echocardiography (STE) is increasingly used during functional assessments. However, reproducibility and dependence on echocardiographic image quality for speckle tracking stress echocardiography in pediatric patients have not been studied to date. 127 consecutive normotensive children without structural heart disease (mean age 13.4 ± 3.0 years, 50.4% female) underwent a stepwise semisupine cycle ergometric protocol. Left ventricular (LV) myocardial peak strain and strain rate were assessed at rest and during exercise. Interobserver and intraobserver assessments were performed and analyzed regarding echocardiographic image quality. LV peak global strain and strain rate were well reproducible with narrow limits of agreement without any significant bias both at rest and during all stages of exercise testing. Moreover, strain rate reproducibility slightly deteriorated in values between −1.5 and −3 s^−1^. Surprisingly, there was no significant difference in reproducibility between optimal, intermediate and poor quality of echocardiographic images. STE derived strain and strain rate measurements in children are feasible and highly reproducible during semisupine cycle ergometric stress echocardiography. Echocardiographic image quality does not seem to influence strain (rate) reproducibility. Myocardial deformation measurements in images with suboptimal visualization quality must be interpreted with caution.

## Introduction

Speckle tracking echocardiography (STE) is an advanced echocardiographic methodology for the non-invasive determination of myocardial performance parameters, namely strain and strain rate^1^. It can be used to identify early subclinical cardiac dysfunction in asymptomatic patients with chronic disease and unremarkable conventional echocardiography such as adult patients with arterial hypertension^2^ and children with type 1 diabetes mellitus^3^. Moreover, STE is sensitive enough to detect even discretely altered cardiac mechanics due to transient changes in blood sugar levels^4^. Furthermore, STE was utilized to demonstrate subclinical myocardial alterations due to primarily non-cardiac diseases like inflammatory bowel disease in both adult and pediatric patient populations^5,6^.

Recently, in a pursuit to increase the diagnostic yield of functional cardiac imaging, STE is increasingly used in combination with dobutamine or ergometer stress testing^7^. The mainstay of this combination in adult medicine is non-invasive workup for coronary artery disease^8^. In pediatric cardiology, strain imaging has been implemented into clinical practice, e.g. to monitor cardiotoxicity in chemotherapy patients^9^. Recently, the hybrid use of speckle tracking imaging and stress echocardiography in children has become a promising method to unmask potential cardiac dysfunction that might remain occult at rest^10^. However, in contrast to diagnostic procedures in adult patients, accurate echocardiographic image acquisition in children is oftentimes more challenging. This is partly due to limited compliance during long-lasting examinations, a lack of understanding of the significance of a diagnostic procedure or a low threshold for personal discomfort. Nevertheless, reproducibility and the significance of image quality during pediatric bicycle ergometer stress testing are still poorly studied. This is the first study to assess the relation of echocardiographic image quality and inter- and intraobserver reproducibility of STE in combination with stress echocardiography in children.

## Methods

### Study population

We enrolled 127 children in this study. Inclusion criteria were good physical health and a written consent by the child itself and by the legal guardian(s). Primary exclusion criteria were the presence of any debilitating symptoms such as fever, pain, fatigue or other past or present health conditions likely affecting physical fitness or the cardiovascular system. This included but was not limited to kidney disease, LV dysfunction, acquired valvular disease, congenital heart disease, developmental delay, body mass index >30 kg/m^2^, pathologic ECG-changes as well as technical limitations such as short leg length or submaximal effort during exercise testing. 15 patients were excluded from the study during the echocardiographic examination due to several reasons: inadequate cycling effort (n = 2), heart disease (n = 1) or insufficient echocardiographic image quality (n = 12).

Initially, all study subjects were assessed with a thorough medical history and physical examination followed by ECG and standard echocardiography. Prior to enrollment, every child as well as their legal guardian had signed a written informed consent. The study sample size of 127 participants was achieved through assumption of power analysis under the consideration of the necessary number of children to form a representative random sample on the one hand as well as keeping the total participants number manageable for a single-center study, on the other hand. A priori, a study design was established sub-categorizing the study cohort into groups of flawless, near-optimal and sub-optimal echocardiographic image quality.

The study was carried out in accordance with the declaration of Helsinki’s ethical principles for medical research involving human subjects and approved by the Witten/Herdecke University ethics committee (*clinical trial number: 103/2014*).

### Conventional echocardiography

We performed a comprehensive echocardiographic study including spectral and color flow Doppler examination in accordance with the standard guidelines of the American Heart Association in all included study participants^11^. The commercially available ultrasound device iE33 by Phillips Ultrasound Inc., USA, with a S5–1 Sector Array transducer (Sector 1–5 MHz) was used. All images were digitally recorded and subsequently transferred to an offline workstation for later analyses, using XCelera Version 3.1.1.422 by Phillips Ultrasound Inc., USA. Images were acquired in the apical 4-, 3- and 2-chamber views, the parasternal long axis view and in two short axis views at the mitral level and at the level of the papillary muscles. M-mode images were obtained at the level of the aortic valve and the LV for subsequent measurement of aortic root diameter, left atrial diameter, interventricular septum, LV cavity and LV posterior wall. Fractional shortening, LV mass, relative wall thickness, LV enddiastolic/endsystolic volume, ejection fraction (EF), stroke volume and cardiac output were assessed. EF was calculated using the modified Simpson’s biplane method. Utilizing PW-Doppler and PW-TDI E/A-ratio, E/E′-ratio and mitral deceleration time were detected for the assessment of LV diastolic function as described elsewhere^12^. All echocardiographic parameters were evaluated utilizing Z-scores^13^.

### Speckle tracking echocardiography

Standard cross-sectional 2D grayscale LV images were acquired for myocardial deformation (strain and strain rate) analyses. Using conventional B-Mode imaging longitudinal strain and strain rate were measured in standard apical 4-chamber (AP4), 3-chamber (AP3) and 2-chamber (AP2) views as previously described in detail^14^. Specifically, circumferential strain (CS) was measured in the standard parasternal short-axis at the mitral valve plane (SAXB) and the papillary muscle plane (SAXM). As recently suggested, 3–4 consecutive cardiac cycles synchronized to a continuous ECG were recorded with frame rate adjusted between 60 and 90 frames per second^15,16^. To achieve accurate deformation parameters, a special focus was set to avoid noise and minimize artifacts during the entire process of echocardiographic image acquisition. Data was anonymized, digitally stored in DICOM format and transferred to an off-line workstation for postprocessing utilizing the commercially available software QLAB Version 10. Global and segmental strain and strain rate were assessed in seven segments per view for longitudinal strain (LS) and six segments for CS by semi-automated tracing of the endocardial border line at end-diastole. Tissue tracking quality was verified in real-time and full thickness coverage of the myocardium including the endocardial and epicardial contours was readjusted manually where necessary. A representative echocardiographic image example of STE assessment in a healthy child during bicycle stress testing is given in Figure S1.

### Stress echocardiography

**Figure 1:**
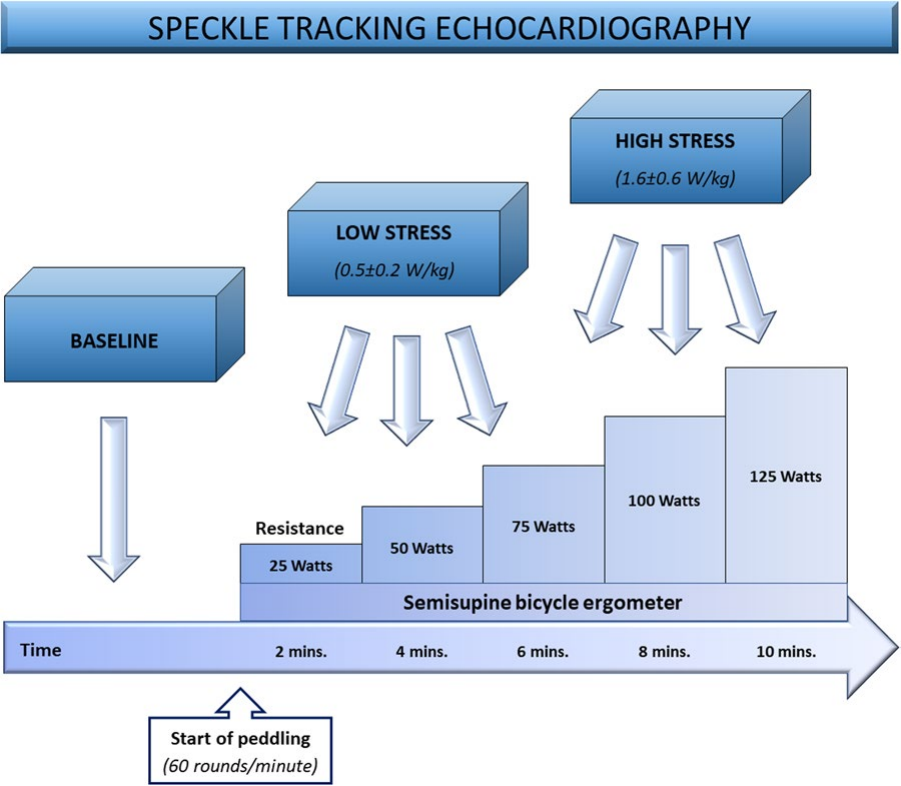
Schematic diagram outlining the experimental setup of speckle tracking echocardiography at baseline and during bicycle ergometer stress testing.

To unmask potential abnormalities in myocardial performance that might remain undiscovered at rest, and to evaluate STE reproducibility during stress testing, we additionally exposed the study population to bicycle ergometer stress testing and performed STE. A schematic diagram outlining the experimental setup of STE application during stress testing is provided in Fig. 1. After completion of resting echocardiography image acquisition, the children were asked to start peddling in a semisupine position on a standard bicycle ergometer at 60 rounds per minute. We utilized a standard ramp protocol with inclining resistance (25 Watts every 2 minutes). Echocardiographic images for STI analysis were acquired at baseline and at two different stress levels: at an intermediate level (approximately 0.5–1 Watt per kilogram body weight) and at the maximum level of physical exhaustion (approximately 1.5–2 Watts per kilogram body weight). A standardized pattern of consecutive images was acquired at all three levels in the above-mentioned viewing planes (SAXB, SAXM, AP4, AP2, and AP3). Peripheral blood pressure and heart rate measurements were obtained at 2-minute intervals and a 3-channel ECG was continuously monitored.

### Image quality scoring

A previously published score of “0”–“3” based on myocardial wall visualization was used by a trained echocardiographer to rate echocardiographic image quality^17^. A score of “3” was assigned to images with >95% myocardial wall visualization, “2” was given when 70–95% of the relevant wall structures were visualized, “1” referred to images of <70% wall visibility. A score of “0” referred to images without clear endocardial border delineation or absent views.

### Assessment of reproducibility

Inter- and intraobserver variabilities were assessed by additional evaluation of baseline and exercise echocardiographic images by a second, independent interpreter, who was blinded to the results of the first echocardiographic reader. In detail, two experienced echocardiographers performed the exact same procedure measuring peak LV strain and strain rate values in all five viewing planes as specifically described above. Baseline and stress test images were assessed this way in all study subjects in a blinded manner. To determine intraobserver variability, one echocardiographer repeated the measurements in a randomly selected order with a minimum of 48 hours between the two analyses. Here, circumferential strain and strain rate were measured in the SAXM view and longitudinal strain and strain rate were repeatedly assessed in the apical 4- chamber view.

### Biostatistical analyses

Demographics, clinical parameters, hemodynamics and echocardiographic data were described as mean and standard deviation. Clinical, hemodynamic and echocardiographic data of the three groups were compared utilizing the ANOVA test. P-values <0.05 constituted statistical significance. The data distribution was graphically displayed using Bland-Altman and correlation graphs. Linear regression analyses and Pearson’s correlation was performed to assess the potential association of echocardiographic variables and clinical parameters. Stata Version 13 (College Station, TX) and Microsoft Excel Version 16.0 for PC were used for all statistical analyses.

### Ethics approval and consent to participate

The study was carried out in accordance with the declaration of Helsinki’s ethical principles for medical research involving human subjects and approved by the Witten/Herdecke University ethics committee (*clinical trial number: 103/2014*).

### Consent for publication

Consent for publication was obtained from all included children, where applicable, as well as from their parents or legal guardians.

### Availability of data and material

The datasets used and/or analyzed during the current study are available from the corresponding author on reasonable request.

## Results

### Patient characteristics

127 children were included in this prospective study. Mean age ( ± standard deviation) was 13.4 ± 3.0 years and gender distribution was almost equal (50.4% female). All included study subjects had no signs of structural heart disease, and their anthropometric data was in accordance with their age and maturation level as evaluated by Z-scores. Mean tanner puberty stage was 2.83 ± 1.25, mean height was 159.3 ± 16.2 cm and mean body weight was 52.6 ± 17.6 kg. On average, the children participated in physical activity 1–2 times per week (Table S1). For all patients the mean heart rate, systolic and diastolic blood pressure at rest were 73 ± 11 beats/min, 111 ± 12 mmHg and 65 ± 9 mmHg, respectively. The mean heart rate increased to 105 ± 11 beats/min at low stress (0.5 ± 0.2 W/kg body weight) and 148 ± 17 beats/min at the maximal stress level (1.6 ± 0.6 W/kg body weight).

### Conventional echocardiographic parameters

Conventional echocardiographic parameters were all normal as evaluated by Z-scores (Table S2)^18^. Specifically, mean LA/Ao ratio was 1.02 ± 0.17, mean estimated LV mass was 121.0 ± 43.5 g and mean LV stroke volume was calculated as 65.9 ± 26.0 ml. The conventional echocardiographic systolic performance indices fractional shortening and LV ejection fraction were 34.43 ± 3.9% and 61.0 ± 4.0%, respectively. Mean mitral annular plane systolic excursion (MAPSE) was 1.51 ± 0.17 cm. Diastolic function was normal as determined by mean E/A ratio of 1.85 ± 0.35 and E/E′ ratio of 8.23 ± 1.44.

### Speckle tracking stress echocardiography

Peak LV global strain and strain rate were detected in all standard echocardiographic planes as specified above. Mean values and standard deviations are indicated for each individual echocardiographic viewing plane and for global strain and global strain rate in the resting state as well as under low and high resistance during exercise testing. As expected, peak LV strain rate was significantly increasing with inclining cycling resistance both longitudinal (−1.36 ± 0.38 vs. −1.54 ± 0.38 vs. −1.83 ± 0.5 s^−1^) and circumferential (−1.64 ± 0.38 vs. −1.79 ± 0.45 vs. −1.79 ± 0.45 s^−1^) throughout all echocardiographic viewing planes (p < 0.01, Figure S2, Table S3). Peak LV longitudinal strain did not differ significantly between the various stress levels (Table S4).

### Strain and strain rate inter- and intraobserver reproducibility at rest and during stress testing

**Figure 2:**
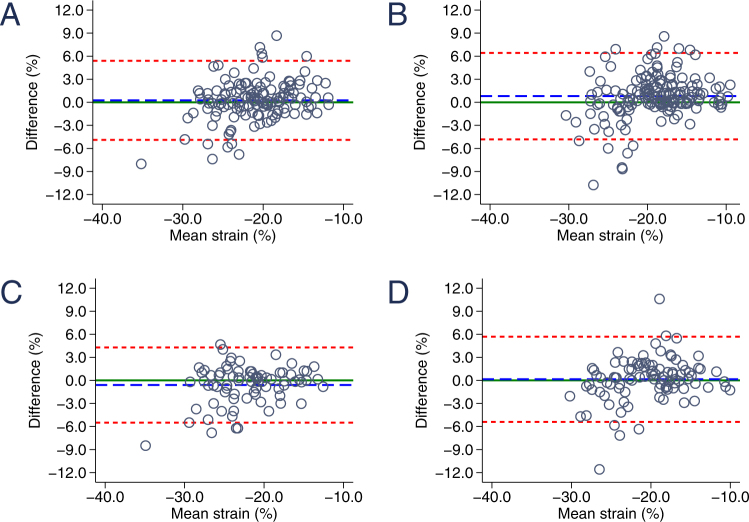
Bland-Altman graphic demonstrating the reproducibility of circumferential strain. (**A**) Interobserver variability of circumferential strain at baseline. (**B**) Interobserver variability of circumferential strain during exercise testing. (**C**) Intraobserver variability of circumferential strain at baseline. (**D**) Intraobserver variability of circumferential strain during exercise testing. The blue dashed line indicates the mean bias (mean difference) and the red dashed lines represent the limits of agreement.

**Figure 3:**
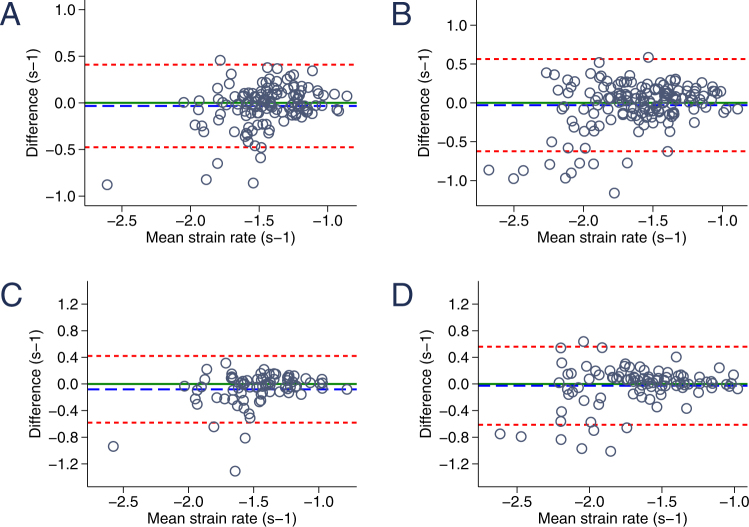
Bland-Altman graphic: reproducibility of circumferential strain rate. (**A**) Interobserver variability of circumferential strain rate at baseline. (**B**) Interobserver variability of circumferential strain rate during exercise testing. (**C**) Intraobserver variability of circumferential strain rate at baseline. (**D**) Intraobserver variability of circumferential strain rate during exercise testing. The blue dashed line indicates the mean bias (mean difference) and the red dashed lines represent the limits of agreement.

**Figure 4:**
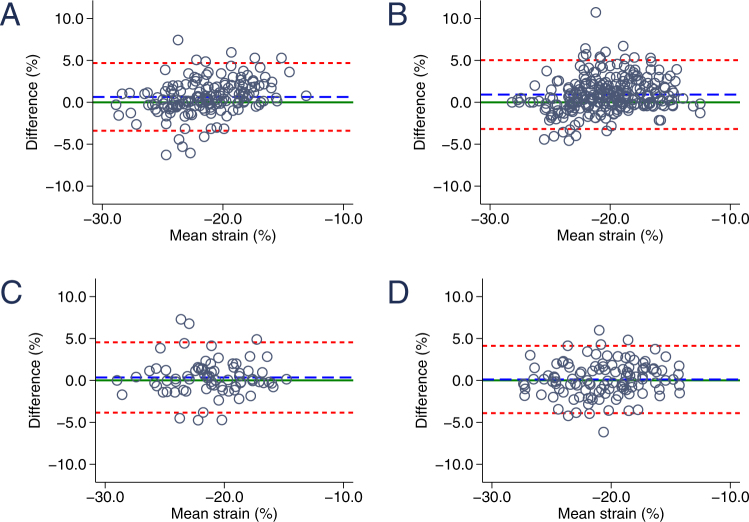
Bland-Altman graphic: reproducibility of longitudinal strain. (**A**) Interobserver variability of longitudinal strain at baseline. (**B**) Interobserver variability of longitudinal strain during exercise testing. (**C**) Intraobserver variability of longitudinal strain at baseline. (**D**) Intraobserver variability of longitudinal strain during exercise testing. The blue dashed line indicates the mean bias (mean difference) and the red dashed lines represent the limits of agreement.

**Figure 5:**
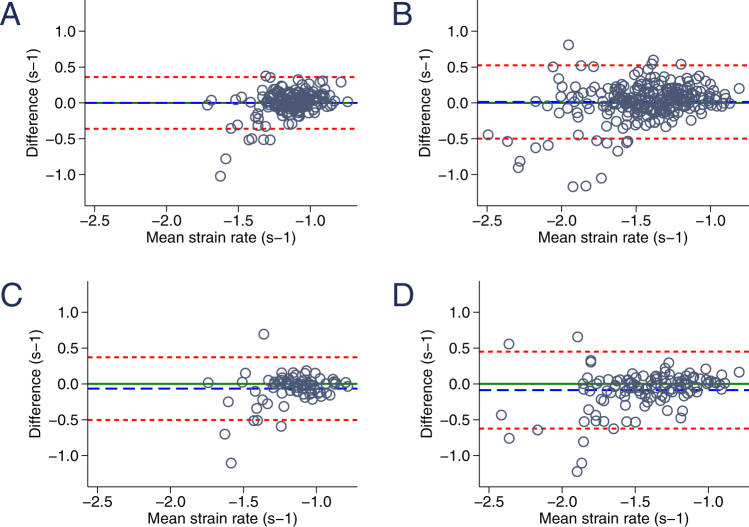
Bland-Altman graphic: reproducibility of longitudinal strain rate. (**A**) Interobserver variability of longitudinal strain rate at baseline. (**B**) Interobserver variability of longitudinal strain rate during exercise testing. (**C**) Intraobserver variability of longitudinal strain rate at baseline. (**D**) Intraobserver variability of longitudinal strain rate during exercise testing. The blue dashed line indicates the mean bias (mean difference) and the red dashed lines represent the limits of agreement.

Overall inter- and intraobserver reproducibility for LV global longitudinal strain and strain rate at rest and during exercise testing are given numerically in Tables 1 and 2 and as Bland-Altman graphs in Figs 2–5. The latter include mean difference of the average values and limits of agreement for inter- and intraobserver variability for circumferential strain (Fig. 2), circumferential strain rate (Fig. 3), longitudinal strain (Fig. 4) and longitudinal strain rate (Fig. 5) at baseline and during stress testing. The mean difference represents bias. A difference of 0% would indicate complete agreement and a bias of more than 1.5% strain was considered significant.

**Table 1:** Inter- and intraobserver reproducibility of global circumferential and global longitudinal *strain* at rest and during exercise testing.

	Global circumferential strain			Global longitudinal strain		
	Baseline	Low stress	High stress	Baseline	Low stress	High stress
Interobserver						
Mean difference	0.38 ± 2.73	0.77 ± 3.12	0.86 ± 2.46	0.63 ± 2.10	0.90 ± 2.07	0.94 ± 2.10
Absolute difference	1.96 ± 1.93	2.29 ± 2.24	1.78 ± 1.90	1.58 ± 1.52	1.61 ± 1.58	1.72 ± 1.52
rho	0.82	0.80	0.84	0.80	0.81	0.79
95%-CI	−0.20; 0.71	0.09; 1.41	0.27–1.46	0.32; 0.96	0.54; 1.23	0.57; 1.37
LOA	−4.89; 5.40	−5.43; 6.93	−3.96; 5.69	−3.40; 4.68	−3.19; 4.96	−3.19; 5.13
Intraobserver						
Mean difference	−0.60 ± 2.50	0.05 ± 3.14	0.34 ± 2.33	0.34 ± 2.14	0.18 ± 1.97	−0.01 ± 2.13
Absolute difference	1.80 ± 1.82	2.06 ± 2.36	1.80 ± 1.49	1.48 ± 1.57	1.57 ± 1.18	1.61 ± 1.38
rho	0.86	0.79	0.87	0.79	0.84	0.81
95%-CI	−1.16;−0.03	−0.80; 0.89	−0.46; 1.04	−0.14; 0.83	−0.31; 0.65	−0.61; 0.64
LOA	−5.49; 4.30	−6.11; 6.20	−4.30; 4.89	−3.85; 4.54	−3.71; 4.05	−4.21; 4.25

**Table 2:** Inter- and intraobserver reproducibility of global circumferential and global longitudinal *strain rate* at rest and during exercise testing.

	Global circumferential strain rate			Global longitudinal strain rate		
	Baseline	Low stress	High stress	Baseline	Low stress	High stress
Interobserver						
Mean difference	−0.04 ± 0.23	−0.05 ± 0.29	0.00 ± 0.31	−0.01 ± 0.19	0.01 ± 0.24	0.03 ± 0.28
Absolute difference	0.16 ± 0.17	0.21 ± 0.22	0.22 ± 0.22	0.12 ± 0.14	0.17 ± 0.17	0.19 ± 0.21
rho	0.69	0.67	0.69	0.56	0.63	0.68
95%-CI	−0.07; 0.01	−0.12; 0.01	−0.07; 0.08	−0.03; 0.03	−0.04; 0.04	−0.02; 0.09
LOA	−0.48; 0.41	−0.63; 0.52	−0.61; 0.62	−0.36; 0.36	−0.48; 0.48	−0.52; 0.58
Intraobserver						
Mean difference	−0.08 ± 0.26	−0.07 ± 0.31	0.03 ± 0.28	−0.07 ± 0.22	−0.10 ± 0.26	−0.08 ± 0.33
Absolute difference	0.16 ± 0.22	0.20 ± 0.24	0.19 ± 0.21	0.13 ± 0.19	0.18 ± 0.22	0.20 ± 0.26
rho	0.69	0.73	0.68	0.53	0.72	0.64
95%-CI	−0.14;−0.02	−0.16; 0.01	−0.05; 0.13	−0.12; −0.01	−0.17;−0.04	−0.14; 0.03
LOA	−0.58; 0.42	−0.67; 0.53	−0.51; 0.59	−0.50; 0.37	−0.62; 0.41	−0.63; 0.51

Global strain agreement was excellent without any significant bias for CS or LS for both inter- and intraobserver variability at rest and throughout all stages of bicycle ergometer stress testing (Table 1). Interestingly, global strain agreement does not significantly deteriorate but remains stable (interobserver) or even increases (intraobserver) in measurements during stress testing (mean CS difference 0.38 ± 2.73 vs. 0.86 ± 2.46 and −0.60 ± 2.50 vs. 0.34 ± 2.33). The second examiner obtained modestly higher circumferential and longitudinal strain values compared to the first examiner as reflected by nonsignificant bias of 0.4 and 0.6%, respectively. Intraobserver variability was equally low with comparably strong correlations and minor bias ranging from −0.6 to 0.3%. The clear majority of all CS and LS measurement repetitions range within the 95% confidence interval (Figs 2 and 4, Table 1).

Strain rate measurements were equally well reproducible (Table 2). Overall interobserver agreement was high with only marginal bias (mean difference for global CSR −0.04 ± 0.23 and LSR −0.01 ± 0.19). During exercise testing interobserver bias was comparably small (mean difference for global CSR 0.00 ± 0.31 and LSR 0.03 ± 0.28) with slightly increased limits of agreement (−0.48; 0.41 vs. −0.61; 0.62 for CSR and −0.36; 0.36 vs. −0.52; 0.58 at rest and during stress testing, respectively). Interestingly, agreement was best for values between 0 and −1.5 at all examined viewing planes. In contrast, distortion was increased for strain rate measurements between −1.5 and −3 (Figs 3 and 5). On average, strain rate intraobserver variability was similar to interobserver reproducibility with slightly increased mean difference values (mean difference of CSR −0.08 ± 0.26 and LSR −0.07 ± 0.22) and less pronounced differences of resting and stress testing limits of agreement (CSR −0.58; 0.42 at rest vs. −0.51; 0.59 during stress testing).

### The influence of echocardiographic image quality on speckle tracking reproducibility

**Figure 6:**
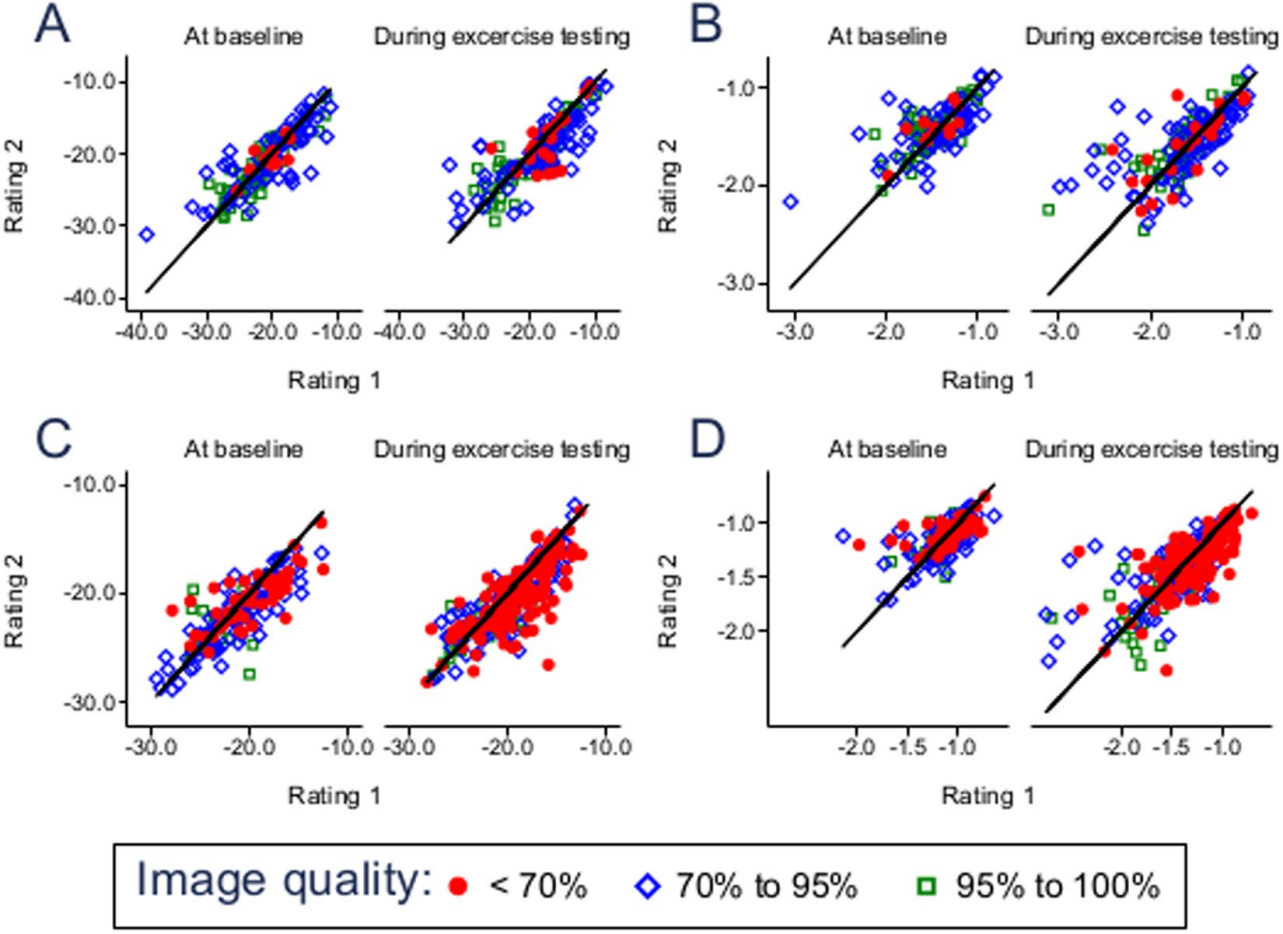
Scatterplots of strain and strain rate measurement inter-rater variability subject to echocardiographic image quality. (**A**) Circumferential strain. (**B**) Circumferential strain rate. (**C**) Longitudinal strain. (**D**) Longitudinal strain rate.

**Figure 7:**
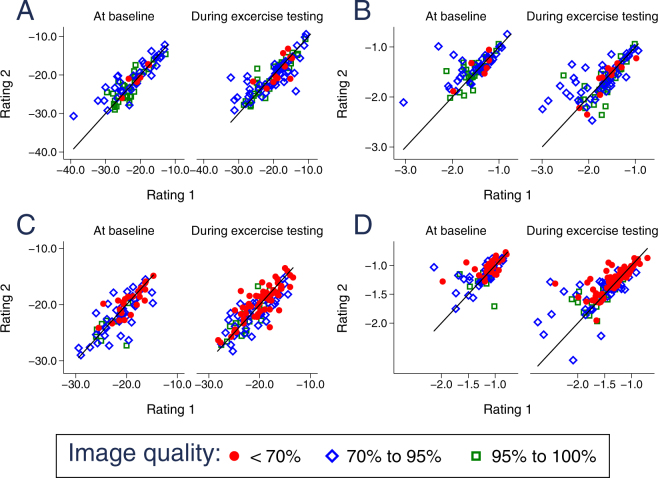
Scatterplots of strain and strain rate measurement intra-rater day-to-day variability subject to echocardiographic image quality. (**A**) Circumferential strain. (**B**) Circumferential strain rate. (**C**) Longitudinal strain. (**D**) Longitudinal strain rate.

To analyze the impact of echocardiographic image quality on the reproducibility of strain and strain rate measurements, we compared interobserver (Fig. 6) and intraobserver (Fig. 7) agreement for optimal (95–100% accurate myocardial wall visualization), suboptimal (70–95% visualization) and limited (<70% visualization) quality of echocardiographic images both in the resting state and during ergometer stress testing. Circumferential strain and strain rate measurements were compared at the levels of the papillary muscles and the mitral anulus. Longitudinal strain and strain rate were assessed at the AP4, AP3 and AP2 viewing planes. Limits of agreement and p-values are indicated both for the total number of analyzed images as well as separately for the three degrees of visualization for strain in Table 3 and for strain rate in Table 4. Importantly, echocardiographic image quality did not significantly influence the degree of reproducibility, neither for strain (Table 3) nor for strain rate measurements (Table 4). Furthermore, there was no systematic difference in limits of agreement between the several levels of image quality (i.e. LOA for 4-chamber derived LSR −0.43; 0.42 in <70% vs. −0.44; 0.41 in 95–100%, p = 0.058). While single p-values fell below the <0.05 border, none was statistically significant after Bonferroni correction for multiple testing.

**Table 3:** Inter-observer reproducibility and the influence of echocardiographic image quality for strain imaging. P-values were calculated using the Kruskal-Wallis-Test and Mann-Whitney-U-Test.

	Stress level	Limits of agreement				p-value			
		total	<70%	70–95%	95% to 100%	total	70% to 95% vs.<70%	95–100% vs. 70% − 95%	<70% vs 95–100%
SAXM	Baseline	−5.74; 5.53	−2.95; 3.74	−7.28; 6.71	−4.25; 4.26	0.044	0.132	0.072	0.375
	Low stress	−6.39; 6.94	−9.16; 9.99	−7.12; 7.98	−4.00; 3.94	0.167	0.635	0.057	0.594
	High stress	−4.82; 6.36	−3.62; 5.06	−5.17; 7.31	−5.17; 6.09	0.775	0.578	0.931	0.462
SAXB	Baseline	−3.41; 4.96	−3.88; 3.54	−4.28; 6.54	−2.17; 3.47	0.450	0.631	0.841	0.687
	Low stress	−3.43; 6.52	−6.62; 12.22	−3.53; 7.31	−2.28; 3.61	0.454	0.623	0.137	0.373
	High stress	−2.31; 4.35	−3.46; 5.78	−2.11; 4.03	−2.05; 4.06	0.253	0.303	0.105	0.477
AP4	Baseline	−2.79; 4.74	−1.99; 4.67	−2.34; 3.96	−6.10; 7.56	0.416	0.101	0.738	0.423
	Low stress	−3.65; 4.90	−3.87; 5.70	−3.91; 4.52	−1.22; 1.68	0.184	0.538	0.045	0.061
	High stress	−3.51; 5.24	−3.96; 5.74	−3.94; 5.50	−1.31; 3.29	0.354	0.610	0.167	0.122
AP 2	Baseline	−3.05; 4.16	−4.08; 4.43	−2.39; 4.06	−3.46; 4.01	0.669	0.446	0.861	0.433
	Low stress	−2.29; 3.65	−2.51; 4.48	−1.64; 2.33	−1.82; 2.14	0.066	0.087	0.704	0.096
	High stress	−2.95; 3.82	−3.01; 4.98	−1.54; 2.18	−4.72; 4.02	0.257	0.074	0.372	0.481
AP 3	Baseline	−4.60; 4.89	−5.62; 5.34	−3.34; 4.60	−6.91; 4.11	0.630	0.705	0.707	1.000
	Low stress	−2.89; 5.98	−3.09; 7.05	−1.92; 3.51	−3.15; 6.23	0.166	0.121	0.265	0.614
	High stress	−2.56; 5.89	−2.92; 6.79	−3.01; 5.33	−1.34; 5.52	0.623	0.782	0.353	0.469

**Table 4:** Inter-observer reproducibility and the influence of echocardiographic image quality for strain rate imaging.

	Stress leve	Limits of agreement				p-value			
		total	<70%	70–95%	95% to 100%	total	70% to 95% vs.<70%	95–100% vs. 70–95%	<70% vs. 95–100%
SAXM	Baseline	−0.57; 0.44	−0.34; 0.28	−0.70; 0.57	−0.44; 0.30	0.035	0.408	0.037	0.376
	Low stress	−0.78; 0.57	−0.71; 0.55	−0.97; 0.67	−0.36; 0.31	0.284	0.505	0.124	0.696
	High stress	−0.55; 0.61	−0.42; 0.52	−0.56; 0.67	−0.63; 0.61	0.706	0.824	0.408	0.668
SAXB	Baseline	−0.30; 0.33	−0.41; 0.26	−0.24; 0.38	−0.32; 0.28	0.085	0.177	0.047	1.000
	Low stress	−0.30; 0.35	−0.18; −0.03	−0.29; 0.41	−0.28; 0.23	0.491	0.743	0.197	0.693
	High stress	−0.70; 0.63	−0.78; 0.67	−0.62; 0.66	−0.81; 0.60	0.655	0.779	0.440	0.439
AP 4	Baseline	−0.47; 0.40	−0.43; 0.42	−0.51; 0.39	−0.44; 0.41	0.179	0.984	0.093	0.058
	Low stress	−0.63; 0.48	−0.34; 0.39	−0.96; 0.49	−0.55; 0.31	0.428	0.257	0.139	0.365
	High stress	−0.67; 0.64	−0.79; 0.75	−0.71; 0.62	−0.13; 0.30	0.643	0.776	0.337	0.541
AP 2	Baseline	−0.23; 0.28	−0.39; 0.32	−0.13; 0.24	−0.15; 0.18	0.349	0.188	0.960	0.248
	Low stress	−0.26; 0.30	−0.25; 0.36	−0.18; 0.17	−0.34; 0.22	0.269	0.254	0.233	0.771
	High stress	−0.48; 0.51	−0.40; 0.53	−0.24; 0.30	−0.94; 0.72	0.135	0.027	0.190	0.920
AP 3	Baseline	−0.24; 0.32	−0.29; 0.27	−0.16; 0.32	−0.40; 0.30	0.299	0.123	0.908	0.711
	Low stress	−0.26; 0.49	−0.26; 0.56	−0.31; 0.48	−0.18; 0.35	0.274	0.591	0.283	0.167
	High stress	−0.25; 0.49	−0.26; 0.46	−0.30; 0.47	−0.16; 0.56	0.367	0.667	0.398	0.298

P-values were calculated using the Kruskal-Wallis-Test and Mann-Whitney-U-Test.

## Discussion

### Speckle tracking echocardiography derived strain and strain rate measurements in children are highly reproducible at rest and during ergometer stress testing

To assess reliability of pediatric STE at baseline and during bicycle stress testing, we analyzed inter- and intraobserver variability in 127 consecutive normotensive children without structural heart disease. Reproducibility was excellent at rest and, interestingly, did not deteriorate during exercise. Since its first steps in the early 2000s, STE has been used in a variety of clinical and experimental scenarios. Today, despite its step-wise implementation in clinical practice of pediatric medicine, i.e. to monitor cardiotoxicity in chemotherapy patients^9^, it is still mostly used for research purposes. Recently, clinical scientists increasingly utilize STE during functional assessments such as dobutamine stress echocardiography^19^ or ergometer stress testing in adult^20^ and pediatric patient populations^21^. However, the reproducibility of these combinatory assessments is still largely unknown, and data is scarce or incomplete in children^22^. Nonetheless, studies in which pediatric STE was analyzed, are mostly in agreement with our results. Firstly, three-dimensional STE derived LV strain was shown to be reproducible in children^23^. Secondly, Wisotzkey *et al*. found global longitudinal strain at rest well reproducible in pediatric heart transplant recipients^24^. Strain rate and reproducibility during stress testing, however, were not assessed in these studies. Thirdly, there is conflicting data on the required level of expertise for the reliable performance of STE dobutamine stress testing. In 41 adult patients, STE was shown to be highly reproducible at all stages of dobutamine stress testing for both expert and novice strain readers^25^. In contrast, STE measurements were highly specific in combination with stress echocardiography in 37 adult patients, but not without a significant degree of dependence on the examiner’s expertise, as reported in another study^26^. One study only assessed longitudinal but not circumferential strain. Moreover, strain rate or image quality were not assessed in both studies and sample size was considerably smaller when compared to the present investigation. Nevertheless, the importance of circumferential strain is not to be underestimated. Recently, Broch and colleagues reported that in patients with compensated aortic regurgitation a relatively high circumferential strain compensates for the reduced longitudinal strain in a manner that is consistent with the preserved EF of these patients^27^.

Importantly, while overall inter- and intraobserver reproducibility was excellent for strain and strain rate imaging, strain rate measurements tended to feature an increased amount of distortion in higher (more negative, to be specific) values between −1.5 and −3 (Figs 3 and 5). This is in line with findings from a similar study in adult patients comparing tissue-Doppler and STE derived strain and strain rate assessments before and during exercise echocardiography^28^. Similarly, Uusitalo and colleagues recently reported increased standard deviations of STE derived global LV strain rate at peak dobutamine stress testing^29^. While only a few studies analyzed STE reproducibility during exercise (mostly in adults), data on strain rate in pediatric stress testing is scarce^30^.

### Echocardiographic image quality does not influence strain and strain rate reproducibility

In a recent well-conducted critical review of current approaches for echocardiographic reproducibility and reliability, the authors found that the statistical metrics used to assess data quality in echocardiographic clinical research vary substantially or were often not reported at all^31^. This is important for two distinct reasons. Firstly, when learning from a published study, the reader should be capable to have an idea of the input raw data quality that is the basis for the subsequently drawn conclusions to facilitate a critical approach to scientific literature. Secondly, when using a diagnostic modality, the clinician must comprehend how to interpret measurements made under suboptimal technical conditions, i.e. echocardiographic noise in STE – a frequent challenge not only in pediatric stress echocardiography. Examples of the latter are abundant in clinical echocardiography. Tissue-Doppler imaging measurements are underestimated with increasing angle-dependency^32^. For the assessment of LV ejection fraction, Simpson biplane method is superior to Teichholz in good image quality and vice versa. On the other hand, bare “eyeballing” is less affected by image quality^33^. Image position errors result in false LV volume quantification which can be overcome with the utilization of increasing numbers of imaging planes, i.e. three-dimensional echocardiography^34^. However, little is known about the STE derived strain and strain rate assessment in suboptimal imaging conditions, neither in adult nor in pediatric patient populations.

To close this gap, we assessed the impact of echocardiographic image quality on inter- and intraobserver STE reproducibility. Remarkably, we found that strain and strain rate measurements did not depend on echocardiographic image quality. This is surprising, as for instance three-dimensional STE derived LV volume measurements have been shown to depend on image quality^35^. On the one hand, limited echocardiography with poor echocardiographic image quality in patients who underwent cardiac surgery has been shown to be of use for the determination of crude measurements such as hemodynamic state^36^. For the three-dimensional echocardiographic LV volume assessment of unselected patients, as little as >60% endocardial border visualization was found to result in reliable measurements^37^. STE on the other hand requires visualization of speckles of each wall aspect to be analyzed. This naturally implies the necessity of optimal image quality. Some authors suggest restricting speckle tracking imaging to subjects with adequate imaging conditions to guarantee favorable accuracy and reproducibility^38^. Yet, our data suggest, that this is not strictly necessary.

### Limitations

The surprising finding that STE reproducibility was not significantly affected by echocardiographic image quality in this study must not be over-interpreted. While the data supporting this result are robust, the study was not designed to evaluate for underlying causes of (un)altered STE accuracy in various image quality scenarios. Possibly, the underlying algorithms of STE post-processing software systems may extrapolate data to an extent, that may lead to false positive assumptions for strain and strain rate measurements of wall aspects that evade thorough tissue tracing due to inadequate image quality. For example, if only 4 out of 6 wall segments are well visualized in a given echocardiographic plane, deformation of the remaining 2 segments with substandard myocardial wall visualization may be corrupted. Clearly, if a segment is not visualized at all or moving out of the echocardiographic viewing plane during a cardiac cycle, it must be excluded from STI analysis. It is the grey area in between, that is challenging (i.e. echocardiographic images with mostly visible myocardium but minor endocardial blurring, etc.). Currently, there is no data to support a guideline on how to proceed in these cases. Therefore, we would recommend excluding substandard images from STI assessments until validation data of STI measurements in impaired echocardiographic image quality is available. Moreover, future studies should investigate difficult imaging scenarios (i.e. children, obese patients, etc.) and correlate STI strain and strain rate to cardiac MRI, taking into consideration echocardiographic intervendor consistency and different underlying post-processing algorithms. Experimental studies may utilize a validation with sonomicrometry^39^, the current gold standard for myocardial deformation imaging to validate STI accuracy in suboptimal echocardiographic image quality.

## Conclusion

STE derived LV strain and strain rate measurements in healthy children feature excellent inter- and intraobserver reproducibility both at baseline and during ergometer stress testing. Importantly, substandard echocardiographic image quality does not influence speckle tracking reproducibility or result in obviously implausible strain (rate) values. Consequently, caution must be paid to critically review STE derived deformation values in suboptimal visualization conditions.

## Electronic supplementary material

Supplementary material

## References

[1] Marcucci C, Lauer R, Mahajan A (2008). New echocardiographic techniques for evaluating left ventricular myocardial function. Semin. Cardiothorac. Vasc. Anesth..

[2] Hensel KO, Jenke A, Leischik R (2014). Speckle-tracking and tissue-Doppler stress echocardiography in arterial hypertension: a sensitive tool for detection of subclinical LV impairment. BioMed research international.

[3] Hensel KO (2016). Subclinical Alterations of Cardiac Mechanics Present Early in the Course of Pediatric Type 1 Diabetes Mellitus: A Prospective Blinded Speckle Tracking Stress Echocardiography Study. J Diabetes Res.

[4] Hensel KO, Grimmer F, Jenke AC, Wirth S, Heusch A (2015). The influence of real-time blood glucose levels on left ventricular myocardial strain and strain rate in pediatric patients with type 1 diabetes mellitus - a speckle tracking echocardiography study. BMC Cardiovasc. Disord..

[5] Kivrak T (2016). Two-dimensional speckle tracking echocardiography is useful in early detection of left ventricular impairment in patients with Crohn’s disease. Eur. Rev. Med. Pharmacol. Sci..

[6] Hensel KO (2017). Speckle Tracking Stress Echocardiography Uncovers Early Subclinical Cardiac Involvement in Pediatric Patients withInflammatory Bowel Diseases. Sci. Rep..

[7] Ilardi F (2016). Quantitative detection of inducible ischemia during dobutamine stress by speckle tracking echocardiography: A dream comes true. Int. J. Cardiol..

[8] Rumbinaite E (2016). Early and late diastolic strain rate vs global longitudinal strain at rest and during dobutamine stress for the assessment of significant coronary artery stenosis in patients with a moderate and high probability of coronary artery disease. Echocardiography.

[9] Tran JC, Ruble K, Loeb DM, Chen AR, Thompson WR (2016). Automated Functional Imaging by 2D Speckle Tracking Echocardiography Reveals High Incidence of Abnormal Longitudinal Strain in a Cohort of Pediatric Oncology Patients. Pediatr. Blood Cancer.

[10] Mese T (2017). Global Deformation Parameters Response to Exercise in Adolescents with Repaired Tetralogy of Fallot. Pediatr. Cardiol..

[11] Lopez L (2010). Recommendations for quantification methods during the performance of a pediatric echocardiogram: a report from the Pediatric Measurements Writing Group of the American Society of Echocardiography Pediatric and Congenital Heart Disease Council. J. Am. Soc. Echocardiogr..

[12] Nagueh SF (2009). Recommendations for the evaluation of left ventricular diastolic function by echocardiography. Eur. J. Echocardiogr..

[13] Chubb H, Simpson JM (2012). The use of Z-scores in paediatric cardiology. Ann. Pediatr. Cardiol..

[14] Hensel, K. O., Wilke, L. & Heusch, A. Transthoracic Speckle Tracking Echocardiography for the Quantitative Assessment of Left Ventricular Myocardial Deformation. *Journal of visualized experiments: JoVE*, 10.3791/54736 (2016). 10.3791/54736 PMC5092220 27805591

[15] Gorcsan J, Tanaka H (2011). Echocardiographic assessment of myocardial strain. J. Am. Coll. Cardiol..

[16] Rosner A (2015). The influence of frame rate on two-dimensional speckle-tracking strain measurements: a study on silico-simulated models and images recorded in patients. Eur. Heart J. Cardiovasc. Imaging.

[17] Johri AM, Chitty DW, Hua L, Marincheva G, Picard MH (2015). Assessment of image quality in real time three-dimensional dobutamine stress echocardiography: an integrated 2D/3D approach. Echocardiography.

[18] Colan, S. D. Normal echocardiographic values for cardiovascular structures (Echocardiography in Pediatric and Congenital Heart Disease). *Wiley-Blackwell, West Sussex, UK* Appendix 1, 765–785 (1999).

[19] Park JH (2016). Layer-specific analysis of dobutamine stress echocardiography for the evaluation of coronary artery disease. Medicine (Baltimore).

[20] Baydar O (2014). Strain analysis during exercise in patients with asymptomatic atrial septal defect. Echocardiography.

[21] Liu, M. Y., Tacy, T., Chin, C., Obayashi, D. Y. & Punn, R. Assessment of Speckle-Tracking Echocardiography-Derived Global Deformation Parameters During Supine Exercise in Children. *Pediatr. Cardiol*., 10.1007/s00246-015-1309-z (2015). 10.1007/s00246-015-1309-z 26671508

[22] Marcus KA (2011). Reference values for myocardial two-dimensional strain echocardiography in a healthy pediatric and young adult cohort. J. Am. Soc. Echocardiogr..

[23] Zhang L (2013). Left ventricular three-dimensional global systolic strain by real-time three-dimensional speckle-tracking in children: feasibility, reproducibility, maturational changes, and normal ranges. J. Am. Soc. Echocardiogr..

[24] Wisotzkey, B. L. *et al*. Feasibility and interpretation of global longitudinal strain imaging in pediatric heart transplant recipients. *Pediatr. Transplant*. **21**, 10.1111/petr.12909 (2017). 10.1111/petr.12909 28295946

[25] Yamada A (2014). Reproducibility of regional and global longitudinal strains derived from two-dimensional speckle-tracking and doppler tissue imaging between expert and novice readers during quantitative dobutamine stress echocardiography. J. Am. Soc. Echocardiogr..

[26] Yang LT (2017). Strain Imaging with a Bull’s-Eye Map for Detecting Significant Coronary Stenosis during Dobutamine Stress Echocardiography. J. Am. Soc. Echocardiogr..

[27] Broch K (2017). Left Ventricular Contraction Pattern in Chronic Aortic Regurgitation and Preserved Ejection Fraction: Simultaneous Stress-Strain Analysis by Three-Dimensional Echocardiography. J. Am. Soc. Echocardiogr.

[28] Lord RN, George K, Jones H, Somauroo J, Oxborough D (2014). Reproducibility and feasibility of right ventricular strain and strain rate (SR) as determined by myocardial speckle tracking during high-intensity upright exercise: a comparison with tissue Doppler-derived strain and SR in healthy human hearts. Echo Res Pract.

[29] Uusitalo V (2016). Two-Dimensional Speckle-Tracking during Dobutamine Stress Echocardiography in the Detection of Myocardial Ischemia in Patients with Suspected Coronary Artery Disease. J. Am. Soc. Echocardiogr..

[30] Leitman M (2017). Speckle Tracking Imaging in Normal Stress Echocardiography. J. Ultrasound Med..

[31] Crowley AL (2016). Critical Review of Current Approaches for Echocardiographic Reproducibility and Reliability Assessment in Clinical Research. J. Am. Soc. Echocardiogr..

[32] Heimdal A, Stoylen A, Torp H, Skjaerpe T (1998). Real-time strain rate imaging of the left ventricle by ultrasound. J. Am. Soc. Echocardiogr..

[33] Grossgasteiger M (2014). Image quality influences the assessment of left ventricular function: an intraoperative comparison of five 2-dimensional echocardiographic methods with real-time 3-dimensional echocardiography as a reference. J. Ultrasound Med..

[34] Chukwu EO (2008). Relative importance of errors in left ventricular quantitation by two-dimensional echocardiography: insights from three-dimensional echocardiography and cardiac magnetic resonance imaging. J. Am. Soc. Echocardiogr..

[35] Kawamura R (2014). Feasibility of left ventricular volume measurements by three-dimensional speckle tracking echocardiography depends on image quality and degree of left ventricular enlargement: validation study with cardiac magnetic resonance imaging. J. Cardiol..

[36] Canty DJ (2017). Assessment of Image Quality of Repeated Limited Transthoracic Echocardiography After Cardiac Surgery. J. Cardiothorac. Vasc. Anesth..

[37] Tighe DA (2007). Influence of image quality on the accuracy of real time three-dimensional echocardiography to measure left ventricular volumes in unselected patients: a comparison with gated-SPECT imaging. Echocardiography.

[38] Nesser HJ (2009). Quantification of left ventricular volumes using three-dimensional echocardiographic speckle tracking: comparison with MRI. Eur. Heart J..

[39] Urheim S, Edvardsen T, Torp H, Angelsen B, Smiseth OA (2000). Myocardial strain by Doppler echocardiography. Validation of a new method to quantify regional myocardial function. Circulation.

