# Supplementary material for: Speckle tracking stress echocardiography in children: interobserver and intraobserver reproducibility and the impact of echocardiographic image quality
Source: Sci Rep. 2018 Jun 15;8:9185. doi: 10.1038/s41598-018-27412-2 (PMC6003923; doi:10.1038/s41598-018-27412-2)
Supplement: Supplementary file 1 — Supplementary material [file 41598_2018_27412_MOESM1_ESM.pdf]

Supplementary material

Speckle tracking stress echocardiography in children: interobserver and intraobserver reproducibility and the impact of echocardiographic image quality

Lucia Wilke[1], Francisca E. Abellan Schneyder[1], Markus Roskopf[1], Andreas C. Jenke[2], Andreas Heusch[1] and Kai O. Hensel[1,*]

[1] *HELIOS University Medical Center Wuppertal, Children's Hospital, Center for Clinical & Translational Research (CCTR), Faculty of Health, Center for Biomedical Education & Research (ZBAF), Witten/Herdecke University, Faculty of Health, Germany*

[2] *EKO Children's Hospital, Department of Pediatric Gastroenterology, Oberhausen, Witten/Herdecke University, Faculty of Health, Germany*

Short title: Pediatric speckle tracking stress echo reproducibility

Supplementary Tables

Table S1. Baseline clinical characteristics and hemodynamics of the study population.

	Variable	**Total study population** (n=127)
	**Age** (years)	13.38±2.97
	**Height** (cm)	159.31±16.24
	**Weight** (kg)	52.57±17.61
	**Body surface** (m^2)	1.52±0.32
	**Body mass index** (kg/m^2)	20.16±4.31
	**Exercise routine** (1=in school; 2=<3 times/week; 3=≥3 times/week)	1.72±0.75
	**Tanner stage**	2.83±1.25
	**Female**	50.4 % (n= 64)
	**Male**	49.6 % (n=63)
**Baseline**	**Heart rate** (bpm)	73.16±10.81
	**BP systolic** (mmHg)	110.74±12.24
	**BP diastolic** (mmHg)	64.56±8.62
**Low stress**	**Heart rate** (bpm)	105.40±11.26
	**Level of resistance** (W/kg body weight)	0.47±0.18
**High stress**	**Heart rate** (bpm)	148.34±17.00
	**BP systolic** (mmHg)	133.76±15.87
	**BP diastolic** (mmHg)	74.68±9.29
	**Level of resistance** (W/kg body weight)	1.63±0.55

Table S2. Conventional echocardiographic parameters derived from two-dimensional and Doppler imaging.

	**Total study population**
**LA/AoR**	1.02±0.17
**Fractional shortening** (%)	34.43±3.86
**Interventricular septal end-systolic diameter** (cm)	1.21±0.21
**Interventricular septal end-diastolic diameter** (cm)	0.85±0.27
**LV end-systolic diameter** (cm)	2.80±0.50
**LV end-diastolic diameter** (cm)	4.29±0.56
**LV posterior wall diameter systolic** (cm)	1.27±0.24
**LV posterior wall diameter diastolic** (cm)	0.89±0.34
**LV mass** (g)	121.04±43.52
**End-systolic volume of the left ventricle** (ml)	40.91±16.72
**End-diastolic volume of the left ventricle** (ml)	104.14±41.34
**Ejection fraction** (%)	60.95±4.04
**Stroke volume** (ml)	65.86±25.97
**E-Wave / A-Wave**	1.85±0.35
**Mitral deceleration time** (s)	0.16±0.03
**E/E'** (cm/s)	8.23±1.44
**MAPSE** (cm)	1.51±0.17

Table S3. Speckle tracking derived peak systolic LV strain rate at rest and during stress testing.

	At rest	Low stress	High Stress	p-value
**Global circumferential strain rate** (s^{-1})	-1.64±0.38	-1.79±0.45	-2.04±0.62	<0.001
**Circumferential strain rate (SAXM)** (s^{-1})	-1.65±0.44	-1.76±0.50	-2.00±0.62	<0.001
**Circumferential strain rate (SAXB)** (s^{-1})	-1.62±0.41	-1.83±0.50	-2.12±0.71	<0.001
**Global longitudinal strain rate** (s^{-1})	-1.36±0.38	-1.54±0.38	-1.83±0.50	<0.001
**Longitudinal strain rate (AP4)** (s^{-1})	-1.33±0.37	-1.49±0.40	-1.80±0.53	<0.001
**Longitudinal strain rate (AP2)** (s^{-1})	-1.40±0.54	-1.52±0.39	-1.86±0.54	<0.001
**Longitudinal strain rate (AP3)** (s^{-1})	-1.38±0.38	-1.62±0.48	-1.85±0.59	<0.001

Table S4. Speckle tracking derived peak systolic LV strain at rest and during stress testing.

	At rest	Low stress	High Stress	p-value
**Global circumferential strain** (%)	-21.34±4.03	-20.14±4.15	-19.29±4.10	0.001
**Circumferential strain (SAXM)** (%)	-22.31±4.60	-20.86±4.46	-20.16±4.62	0.003
**Circumferential strain (SAXB)** (%)	-20.32±4.13	-19.53±4.53	-18.54±4.00	0.021
**Global longitudinal strain** (%)	-20.59±2.64	-20.39±2.82	-20.29±3.40	0.859
**Longitudinal strain (AP4)** (%)	-20.66±2.84	-20.55±3.21	-20.72±3.63	0.931
**Longitudinal strain (AP2)** (%)	-20.74±3.28	-20.47±3.10	-20.49±3.66	0.808
**Longitudinal strain (AP3)** (%)	-20.49±3.34	-20.58±3.62	-20.28±3.64	0.848

Supplementary Figures

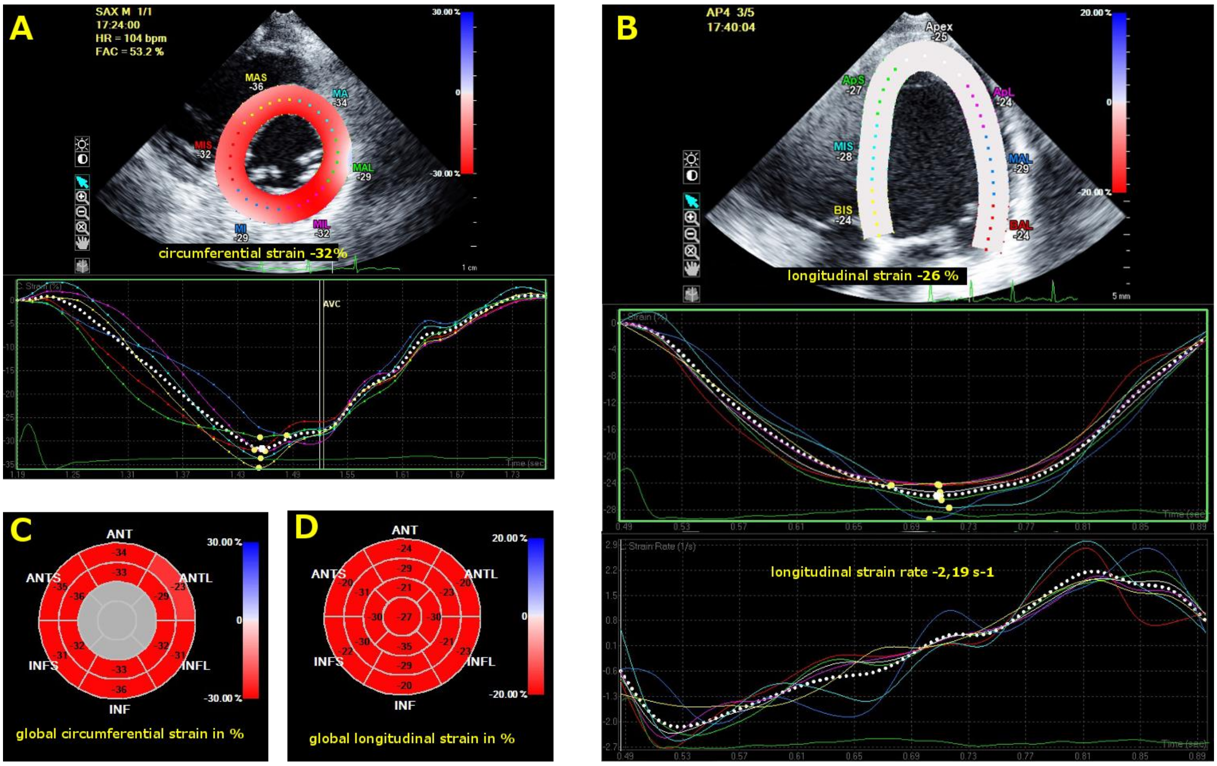

Figure S1. Echocardiographic image examples of LV speckle tracking derived strain and strain rate analyses in a healthy child during bicycle stress testing. **A** Circumferential strain assessment in the short axis at the level of the papillary muscles (top) and respective segmental (colours) and global (white dotted) time-strain curves are shown (bottom). An ECG signal is given below for timing synchronization. **B** Apical 4-chamber view derived longitudinal speckle tracking measurement (top) with respective strain and strain rate time-curves and a synchronized ECG lead below. **C** Bull's-eye view of peak LV global circumferential strain. **D** Bull's-eye view of peak LV global longitudinal strain.

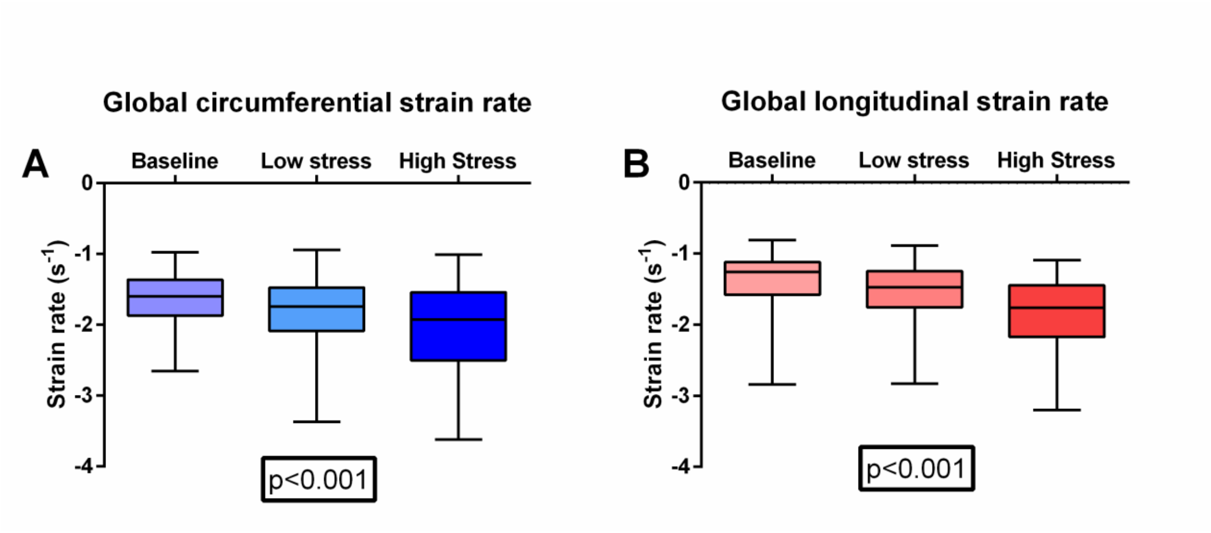

Figure S2. Global circumferential (A) and longitudinal (B) strain rate at rest and during stress testing.
